# Short leukocyte telomere length is associated with obesity in American Indians: The strong heart family study

**DOI:** 10.18632/aging.100664

**Published:** 2014-05-15

**Authors:** Shufeng Chen, Fawn Yeh, Jue Lin, Tet Matsuguchi, Elizabeth Blackburn, Elisa T. Lee, Barbara V. Howard, Jinying Zhao

**Affiliations:** ^1^Department of Epidemiology, School of Public Health and Tropical Medicine, Tulane University, New Orleans, LA 70112, USA; ^2^Department of Evidence Based Medicine and Division of Population Genetics, State Key Laboratory of Cardiovascular Disease, Cardiovascular Institute and Fuwai Hospital, National Center for Cardiovascular Diseases, Chinese Academy of Medical Sciences and Peking Union Medical College, Beijing, China; ^3^Center for American Indian Health Research, University of Oklahoma Health Science Center, Oklahoma City, OK 73104, USA; ^4^Department of Biochemistry and Biophysics, University of California, San Francisco, CA 94143, USA; ^5^MedStar Health Research Institute, Hyattsville, MD 20782, and Georgetown/Howard Universities Center for Clinical and Translational Science, Washington, DC 20007, USA

**Keywords:** obesity, Leukocyte telomere length, American Indians, strong heart family study

## Abstract

Shorter leukocyte telomere length (LTL) has been associated with a wide range of age-related disorders including cardiovascular disease (CVD) and diabetes. Obesity is an important risk factor for CVD and diabetes. The association of LTL with obesity is not well understood. This study for the first time examines the association of LTL with obesity indices including body mass index, waist circumference, percent body fat, waist-to-hip ratio, and waist-to-height ratio in 3,256 American Indians (14-93 years old, 60% women) participating in the Strong Heart Family Study. Association of LTL with each adiposity index was examined using multivariate generalized linear mixed model, adjusting for chronological age, sex, study center, education, lifestyle (smoking, alcohol consumption, and total energy intake), high-sensitivity C-reactive protein, hypertension and diabetes. Results show that obese participants had significantly shorter LTL than non-obese individuals (age-adjusted P=0.0002). Multivariate analyses demonstrate that LTL was significantly and inversely associated with all of the studied obesity parameters. Our results may shed light on the potential role of biological aging in pathogenesis of obesity and its comorbidities.

## INTRODUCTION

Telomeres are specialized nucleotide (TTAGGG)_n_ repeats and their associated proteins on the distal ends of human chromosomes. They are critical in maintaining chromosomal stability during mitotic cell proliferation. Because of the end-replication problem, telomere length shortens progressively during each round of cell division [1]. Telomere length declines gradually with age and has been used as a valuable biomarker for cellular senescence and biological aging. Shorter leukocyte telomere length (LTL) has been associated with a variety of age-related disorders, such as hypertension [2], diabetes [3], cardiovascular diseases (CVD) [4, 5] and certain types of cancers [6, 7].

Obesity is an escalating health problem that is associated with aging and age-related diseases. The prevalence of obesity is reaching epidemic proportions among all ethnic groups including American Indians. Although the precise mechanisms linking obesity to age-related disorders remain largely unknown, it is possible that biological aging driven by telomere shortening plays a central role. In cross-sectional epidemiological studies, shorter LTL has been associated with body mass index (BMI), waist-to-hip ratio (WHR) and visceral fat [8, 9]. In an intervention trial of 521 subjects, reduction in adiposity indices corresponding to a Mediterranean diet intervention was accompanied by increased LTL [10], probably through telomere maintenance [11]. Weight loss induced by calorie-restricted diets was also associated with increased telomere length in rectal mucosa of obese men [12]. However, these findings were primarily derived from European Caucasians in relatively small subgroups of patients or research subjects, and results were inconsistent across study populations [13-17]. Given the potential impact of genetic [18-20] and lifestyle [21, 22] factors on telomeric aging and obesity pathologies, confirmation of previous findings in other ethnic groups with a wide range of age distribution in community-based populations is clearly required.

Obesity and overweight are highly prevalent among American Indians [23], a minority group that may have distinct genetic makeup and lifestyle factors in comparison with other populations [24]. Short LTL has been significantly predictive of type 2 diabetes [3] and carotid atherosclerosis (unpublished data) in this population. The purpose of this study is to examine the possible association of LTL with obesity in American Indians participating in the Strong Heart Family Study (SHFS).

## RESULTS

### Characteristics of the study participants

Among the 3,256 participants (1,941 women; mean age, 38.7 years), 1,871 (57.5%) participants were obese. The prevalence of obesity in AZ (73.7%) was significantly higher than that in OK (52.6%) and DK (46.1%). Women were more likely to be obese (60.6%) than men (52.8%). The prevalence of diabetes and hypertension was 20.6% and 31.3%, respectively. Compared with non-obese individuals, obese individuals were older, had shorter LTL, higher levels of blood pressure, fasting glucose, unfavorable lipid profiles, and higher prevalence of hypertension and diabetes, but obese participants were less likely to be current smokers or drinkers (Table S1).

Clinical characteristics of study participants according to LTL quintiles are shown in Table 1. Compared to participants in the highest quintile of LTL (longest telomere length), those with shorter LTL had higher levels of WC, BMI, WHR, WHtR, and hsCRP after adjustment for age. Prevalence of obesity and diabetes were significantly higher among participants in the lowest LTL quintile than that in the highest quintile. Prevalence of obesity in participants with 1^st^ through 5^th^ quintile of LTL was 61.8%, 62.2%, 60.1%, 56.8% and 46.4% (age-adjusted P for trend <0.0001), respectively. The corresponding prevalence of diabetes was 31.5%, 27.3%, 19.5%, 14.9% and 9.7%, respectively (age-adjusted P for trend <0.0001).

**Table 1:** Characteristics of study participants by LTL quintiles

Variables	Quintile 1	Quintile 2	Quintile 3	Quintile 4	Quintile 5	P for trend*
N	651	651	652	651	651	
LTL (T/S ratio)	0.68±0.11	0.88±0.04	0.99±0.03	1.09±0.04	1.31±0.16	<0.0001*
Age (years)	47.6± 15.9	42.5±15.9	39.1±15.9	34.5±15.2	30.0±14.0	<0.0001
Men, n (%)	271 (41.6)	267 (41.0)	270 (41.4)	241 (37.0)	266 (40.9)	0.009*
Education (years)	12.0±2.3	11.9±2.4	12.0±2.3	11.8±2.3	11.5±2.2	0.10*
Energy intake (kcal)	2586±1764	2530±1777	2661±1826	2996±2806	2727±1907	0.15*
WC (cm)	107.8±17.7	106.4±18.6	104.9±18.5	103.6±17.7	98.0±17.6	<0.0001*
Percent body fat (%)	37.7±10.0	38.0± 9.9	37.3± 9.9	37.9±10.4	35.4±10.8	0.57*
BMI (kg/m^2^)	33.0± 7.6	33.2±8.4	32.5±7.8	32.2±7.5	30.1±7.6	<0.0001*
WHR	0.93±0.08	0.91±0.08	0.91±0.08	0.90±0.08	0.89±0.08	<0.0001*
WHtR	0.65±0.11	0.64± 0.11	0.63±0.11	0.62±0.11	0.58±0.11	<0.0001*
Fasting Glucose (mg/dL)	124.8±62.7	119.0±55.2	113.3±52.3	108.6±50.3	101.0±34.6	0.001*
hsCRP (mg/L)	7.94±9.73	7.43±9.62	6.30±7.52	6.06±7.50	5.04±7.01	<0.0001*
Physical activity (steps/d)	5164±3852	5426±3684	5691±3553	6092±4187	6478±4012	0.40*
Smoking, n (%)	218 (33.8)	207 (31.8)	236 (36.3)	229 (35.2)	224 (34.4)	0.16*
Drinking, n (%)	345 (53.5)	373 (57.4)	378 (58.1)	403 (61.9)	426 (65.4)	0.18*
Hypertension, n (%)	265 (40.7)	246 (37.8)	204 (31.3)	187 (28.8)	116 (17.8)	0.88*
Diabetes, n (%)	205 (31.5)	177 (27.3)	127 (19.5)	97 (14.9)	63 (9.7)	<0.0001*
CVD, n (%)	42 (6.5)	25 (3.8)	21 (3.2)	15 (2.3)	11 (1.7)	0.61*
Obesity, n (%)	402 (61.8)	405 (62.2)	392 (60.1)	370 (56.8)	302 (46.4)	<0.0001*

* Adjusted for age

Table 2 presents the partial correlation coefficients of LTL with obesity parameters and risk factors. LTL was negatively correlated with age (sex-adjusted *r* = −0.35, P<0.0001). After adjustments for both age and gender, LTL remained significantly and inversely correlated with all obesity indices and hsCRP (all P < 0.001).

**Table 2:** Partial correlation coefficient of LTL with obesity traits and related factors (N=3256)

Variables	Correlation coefficient*	P-value
Age	−0.35	<0.0001
Education in years	0.01	0.60
Energy intake	−0.02	0.21
Waist circumference	−0.13	<0.0001
Percent body fat	−0.12	<0.0001
Body mass index	−0.13	<0.0001
Waist-to-hip ratio	−0.11	<0.0001
Waist-to-height ratio	−0.15	<0.0001
High-sensitivity C-reactive protein	−0.09	<0.0001

* Adjusted for age and gender where appropriate

### Association of LTL with adiposity traits

LTL as a continuous variable was inversely associated with all obesity measurements after adjustments for age, sex, study center, education, lifestyle factors, hsCRP, hypertension and diabetes. On average, one unit decrease in LTL was associated with 5.96 cm increase in WC, 1.50% increase in %BF, 2.56 kg/m^2^ increase in BMI, 0.02 unit increase in WHR and 0.04 unit increase in WHtR. Compared to participants whose LTL was in the highest quintile, those in the lowest quintile had significantly higher WC, BMI, WHR and WHtR (all P ≤0.001). Multivariable-adjusted association of LTL with adiposity phenotypes was shown in Table 3.

**Table 3:** Multivariate association of LTL with anthropometric measurements in American Indians*

Variables	LTL	β (95% CI)	P	P for trend
Waist circumference	Continuous	−5.96 (−8.70 − −3.22)	<.0001	
	Q1 vs. Q5	3.92 (2.05 − 5.79)	<.0001	
	Q2 vs. Q5	2.79 (0.93 − 4.65)	0.003	
	Q3 vs. Q5	2.86 (0.88 − 4.85)	0.005	
	Q4 vs. Q5	2.76 (0.78 − 4.75)	0.007	0.0003
Percent body fat	Continuous	−1.50 (−2.74 − −0.26)	0.02	
	Q1 vs. Q5	0.77 (−0.12 − 1.66)	0.09	
	Q2 vs. Q5	0.84 (0.06 − 1.62)	0.04	
	Q3 vs. Q5	0.75 (−0.15 − 1.64)	0.10	
	Q4 vs. Q5	1.04 (0.08 − 1.99)	0.03	0.14
Body mass index	Continuous	−2.56(−3.78 − −1.33)	<.0001	
	Q1 vs. Q5	1.49 (0.62 − 2.36)	0.0009	
	Q2 vs. Q5	1.42 (0.58 − 2.26)	0.001	
	Q3 vs. Q5	1.27 (0.43 − 2.11)	0.003	
	Q4 vs. Q5	1.08 (0.16 − 2.01)	0.02	0.0005
Waist-to-hip ratio	Continuous	−0.023 (−0.032 − −0.014)	<.0001	
	Q1 vs. Q5	0.016 (0.008 − 0.023)	<.0001	
	Q2 vs. Q5	0.006 (−0.001 − 0.013)	0.08	
	Q3 vs. Q5	0.010 (0.003 − 0.017)	0.008	
	Q4 vs. Q5	0.009 (0.002 − 0.016)	0.02	0.002
Waist-to-height ratio	Continuous	−0.038(−0.054 − −0.022)	<.0001	
	Q1 vs. Q5	0.024 (0.013 − 0.036)	<.0001	
	Q2 vs. Q5	0.017 (0.007 − 0.028)	0.002	
	Q3 vs. Q5	0.017 (0.006 − 0.029)	0.004	
	Q4 vs. Q5	0.017 (0.005 − 0.028)	0.007	0.0001

* Adjusted for age, sex, study center, education, smoking, alcohol drinking, and total energy intake, hsCRP, hypertension and diabetes.

In the model treating obesity as a categorical variable (yes/no), participants with shorter LTL (lowest LTL quintile) were more likely to be obese than those with longer LTL (highest LTL quintile, OR=1.39; 95% CI, 1.10-1.76). Results are shown in Table 4. Additional adjustments for physical activity level or batch effect did not change the results. The observed associations also persisted after excluding children from statistical analysis.

**Table 4:** Association of LTL with obesity (yes/no) in American Indians

LTL	OR	95% CI	P value	P for trend
**Model 1**				
Continuous	0.49	0.35 − 0.71	0.0001	
Q1 vs. Q5	1.58	1.23 − 2.02	0.0003	
Q2 vs. Q5	1.58	1.24 − 2.00	0.0002	
Q3 vs. Q5	1.54	1.23 − 1.92	0.0002	
Q4 vs. Q5	1.39	1.08 − 1.80	0.0108	<.0001
***Model 2***				
Continuous	0.49	0.35 − 0.70	<0.0001	
Q1 vs. Q5	1.55	1.22 − 1.97	0.0003	
Q2 vs. Q5	1.53	1.18 − 1.98	0.0014	
Q3 vs. Q5	1.49	1.19 − 1.88	0.0006	
Q4 vs. Q5	1.36	1.04 − 1.78	0.0245	0.0002
**Model 3**				
Continuous	0.56	0.40 − 0.79	0.001	
Q1 vs. Q5	1.39	1.10 − 1.76	0.0078	
Q2 vs. Q5	1.37	1.04 − 1.79	0.0275	
Q3 vs. Q5	1.40	1.11 − 1.78	0.0039	
Q4 vs. Q5	1.27	0.97 − 1.66	0.0794	0.0065

Model 1: adjusted for age, sex, and study center; Model 2: further adjusted for education, smoking, alcohol drinking, and total energy intake; Model 3: additionally adjusted for high-sensitivity C reactive protein, hypertension and diabetes.

## DISCUSSION

In a large, well-characterized population of American Indians, LTL is inversely correlated with all obesity indices. Short LTL was significantly associated with obesity and related adiposity measurements, independent of traditional risk factors including chronological age, lifestyle factors, and obesity-related inflammation or comorbidities, e.g., diabetes and hypertension. Our findings may shed light on the potential mechanisms linking biological aging, obesity, and age-related disorders.

Several previous studies have examined the association of telomere length with obesity, but results were inconsistent, and the relationship between telomere shortening and obesity remains largely inconclusive. In a population of 1,122 white women, obese individuals had significantly shorter LTL than lean women [9]. A recent meta-analysis also revealed an inverse association between LTL and BMI in cross-sectional studies [25]. In a 5-year intervention trial, improvement in obesity indices in response to Mediterranean diet paralleled with increasing telomere length [10]. Further study indicated that the beneficial effects of Mediterranean diet on longevity could likely be determined by telomere maintenance [11]. Weight gain was also accompanied by reduced TL among obese women [8], but the favorable effect of weight loss and exercise on telomere length was unable to be replicated in postmenopausal women [16]. These discrepant results indicate that the relationship between telomere shortening, biological aging and obesity could be very complex.

Inflammation is an important biological mechanism known to be implicated in obesity [26], and telomere shortening has also been associated with inflammation [27]. In our analysis, LTL was negatively correlated with hsCRP, but the associations between telomeres and obesity indices were not substantially attenuated after further adjustment for hsCRP, suggesting that this association may not attribute to obesity-related inflammation. However, it should be noted that, hsCRP might not reflect the entire spectrum of inflammatory processes, although it is one of the most sensitive acute-phase reactant and has been widely used as an indicator of inflammation. In addition, different populations may have different inflammatory profiles, and thus future research should also investigate whether the observed association of telomeres with obesity in our study is independent of other inflammatory biomarkers in different populations. Moreover, the observed association of LTL with obesity withstood additional adjustments for diabetes or hypertension, indicating that this association may not be attributable to these two comorbidities.

Up to date, limited research has investigated the potential biological mechanisms underlying the linkage between accelerated telomere shortening and obesity. A previous study in mice demonstrated that telomere shortening may contribute to metabolic dysfunction through its impact on mitochondrial biogenesis [28]. In a recent experimental study, disruption of *Rap1*, a telomere-binding protein, resulted in a cascade of metabolic dysfunction, and *Rap1*-deficient mice exhibited accumulation of abdominal fat, insulin resistance, and other metabolic abnormalities, revealing a critical role of telomere proteins in body weight homeostasis [29, 30]. The mammalian target of rapamycin (mTOR) pathway may represent another important biological mechanism linking telomeric aging to obesity, because mTOR pathway integrates insulin and nutrient signaling in numerous cell types and enhanced mTOR signaling has been implicated in cellular senescence. [31-33] Moreover, hyperactivity of the mTOR signaling has been associated with obesity and its related traits such as insulin resistance, type 2 diabetes, and cardiovascular diseas [34-37]. Furthermore, rapamycin slows down aging, prevents age-related diseases and extends maximal lifespan in mice [37]. These investigations open new avenues for understanding the potential significance of accelerated biological aging in obesity and other age-related disorders.

Numerous previous studies have reported that indicators of abdominal obesity (e.g., WC, WHtR) are better discriminators of cardiometabolic risk than indices of general obesity (e.g., BMI, %BF) [38-42].Given that accelerated aging may represent a common denominator of obesity and related cardiometabolic comorbidities, it would be interesting to examine whether telomere length differentially influences body fat distribution, and if so, whether this explains the observed differential effect of body fat distribution on cardiometabolic risk. In this large, community-based epidemiological study of American Indians, LTL was inversely associated with all indices of obesity. We did not observe a differential effect of telomeric aging on body fat distribution, though the association of telomere length with abdominal obesity (e.g., WC) appears to be stronger than that with general obesity (e.g., BMI, %BF).

Several limitations of this study deserve mention. First, the cross-sectional nature of our analysis precludes any causal inference. Second, our results were derived from a cohort of American Indians who suffer from high rates of obesity and diabetes, and may also have distinct genetic and lifestyle risk profiles, so generalizability to other populations is unknown. However, given the rising tides of obesity and diabetes in other populations, it is plausible to speculate that our findings could be generalized to the general population. In addition, because many of the participants in our study population were centrally obese, the observed associations need to be confirmed in other cohorts. Finally, although we were able to control many of the known risk factors, we cannot entirely exclude the possibility of confounding by other unknown or unmeasured risk factors. Nonetheless, this is the first study to examine the association between LTL and obesity in a large, population-based study of American Indians. The comprehensive clinical phenotyping, the high quality telomere data, and a careful control for possible confounding factors represent the major strengths of the current investigation.

In summary, LTL was negatively associated with obesity in American Indians participating in the SHFS, independently of chronological age, lifestyle factors and obesity-related inflammation or comorbidities. Findings from this study provide valuable information towards a better understanding of obesity, and also highlight the importance of accelerated biological aging in obesity pathology.

## METHODS

### Study population

The Strong Heart Family Study (SHFS), a component of the Strong Heart Study (SHS), is a multicenter, family-based prospective study designed to identify genetic factors for CVD and their risk factors in American Indians. The study was initiated in 2001-2003 by recruiting 3,665 individuals (14-93 years old) from 13 tribes in Arizona (AZ), Oklahoma (OK) and North/South Dakota (DK). Study participants underwent a clinical examination including a personal interview and physical examination. Information on demographic factors, socioeconomic status, medical history, medication use, and lifestyle factors was collected by personal interview using standard questionnaires. A physical examination was conducted, and fasting blood samples were collected for laboratory tests, including fasting glucose, insulin, high-sensitivity C-reactive protein (hsCRP), lipids and creatinine. Study design and methods of the SHFS have been reported previously [43, 44]. The SHFS protocol was approved by the Institutional Review Boards from the Indian Health Service and the participating study centers. All participants gave informed consent.

Among the 3,665 participants, 3,492 participants had completed data of telomere length and anthropometric measurements. Because some conditions may significantly influence body weight, we excluded individuals with cirrhosis of liver (n=50), chronic renal failure (n=55), cancer (n=141) or women who were pregnant at the time of clinical visit (n=5). The remaining 3,256 participants were included in the present investigation.

### Assessments of obesity and related traits

Anthropo-metric measurements included body weight, height, waist circumference (WC) and hip circumference measured with participants wearing light clothing and without shoes by trained study researchers. Body height and weight were measured for each participant in a standing position. WC was measured at the level of the umbilicus while the participant was in a supine position. Hip circumference was measured at the level of widest circumference over greater trochanters with the legs close together in a standing position. BMI was defined as body weight in kilograms divided by the square of the height in meters (kg/m^2^). WHR was calculated as waist circumference divided by hip circumference. Waist-to-height ratio (WHtR) was calculated as waist circumference divided by height. Percent body fat (%BF) was estimated with an RJL impedance meter (Model B14101; RJL Equipment Company, Detroit, MI) using an equation based on total body water [45, 46]. In participants aged 18 years and older (n=2,927), obesity was defined as BMI ≥ 30 kg/m^2^ according to the National Institutes of Health (NIH) guidelines [47]. Among children (<18 years old) (n=329), obesity was defined as BMI ≥ 95^th^ percentile by age and gender, according to the recommended parameters from the National Center for Health Statistics (NCHS) 2000 growth charts [48].

### Assessments of covariates

Education level was collected by standard questionnaires. Education level was coded into three categories (1=less than high school, reference; 2=high school graduate; 3= some college or higher). Cigarette smokers were defined as persons who smoked at least 100 cigarettes during their lifetime. Those who were smoking cigarette at the visit time were classified as current smokers. Current drinkers are those who had consumed any alcohol during the past year. Dietary intake was assessed using the Willett self-administered semi-quantitative foodfrequency questionnaire (FFQ) [49], which collected dietary data for the 12 months prior to testing. Each participant received a pedometer, instructions for wearing the pedometer, and an activity diary at their clinical examination. They were asked to wear the pedometer for seven consecutive days and to record the number of the steps taken daily in the activity diary. Physical activity was assessed by the mean number of steps per day calculated by averaging the total number of steps recorded each day during the 7-day period. Hypertension was defined as blood pressure levels of 140/90 mm Hg or higher or current use of antihypertensive medications [50]. According to the American Diabetes Association criteria, diabetes was defined as fasting plasma glucose ≥ 7.0 mmol/L or receiving insulin or oral hyperglycemic treatment [51].

### Measurement of leukocyte telomere length (LTL)

Genomic DNA from peripheral blood was isolated according to standard methods. LTL, as measured by T/S ratio, was performed by Dr. Elizabeth Blackburn's laboratory at the University of California, San Francisco using an automated, high-throughput telomere length assay system. Detailed lab protocols and methods have been described previously. [3, 20] Briefly, LTL was quantified by qPCR using a serially diluted standard DNA and the standard curve method. The ratio of the telomeric product vs. the single copy gene reflects the average length of the telomeres. A single copy gene was amplified in parallel to normalize the quantity of the input DNA. Each DNA sample was assayed three times and the mean value was used in statistical analysis. For quality control, we included seven control DNA samples from various cancer cell lines in each assay plate. These control samples allowed us to create standard curves, which were then integrated into a composite standard curve used for T and S concentration calculations. Theintra- and inter-assay %CV was 4.6% and 6.9%, respectively. Lab technicians were blinded to any knowledge of clinical data.

### Statistical analyses

Clinical characteristics of the study participants were compared across the quintiles of LTL. Cross-sectional association of LTL with each obesity phenotype (dependent variable) was assessed using linear mixed model, including WC, %BF, BMI, WHR, and WHtR, adjusting for established risk factors for obesity (described below). Linear mixed model was used here to account for the lack of independence among family members. In the association analyses, LTL was first analyzed as a continuous variable, and then categorized into quintiles to facilitate interpretation. Regression coefficient and 95% confidence interval (CI) for each measurement was assessed by constructing a series of hierarchical models adjusting for potential confounders: Model 1, adjusted for age, sex, and study center; Model 2, further adjusted for education and lifestyle factors including smoking, alcohol drinking, and total energy intake; Model 3, additionally adjusted for hsCRP, hypertension and diabetes. The odds ratio (OR) and 95% CI for the association of LTL with obesity (yes/no) were assessed using multivariate logistic regression model implemented in GLIMMIX procedure in SAS. All statistical analyses were done using SAS 9.3 (SAS Institute, Inc., Cary, NC).

Given the close relationship of physical activity with obesity, we additionally adjusted for level of physical activity to examine whether it influences the association between LTL and obesity. To examine whether inclusion of children influences the association of LTL with obesity, we conducted additional analysis by excluding children (aged <18 years, n=329) from statistical analysis. To examine whether and how batch effects influence our results, we conducted additional analysis by further adjusting for batch in the above statistical model.

## SUPPLEMENTARY TABLE


