# Supplementary material for: Short leukocyte telomere length is associated with obesity in American Indians: The strong heart family study
Source: Aging (Albany NY). 2014 May 15;6(5):380–9. doi: 10.18632/aging.100664 (PMC4069265; doi:10.18632/aging.100664)
Supplement: Supplementary file 1 [file aging-06-380-s001.pdf]

SUPPLEMENTARY TABLE

Table S1. Comparison of clinical characteristics between obese and non-obese participants

Variables	Non-obesity	Obesity	P-value
N	1385	1871	
LTL	1.02±0.25	0.97±0.22	0.0003
Age (yrs)	37.9±17.8	39.4±15.5	0.58
Men, n (%)	621(44.8)	694(37.1)	0.001
Education (yrs)	11.8±2.3	11.9±2.3	0.04
Total energy intake (kcal)	2762±2206	2658±1956	0.61
Waist circumference (cm)	89.0±10.3	115.3±14.6	<0.0001
Hip circumference (cm)	101.9±7.4	123.4±13.5	<0.0001
Percent body fat (%)	30.1±8.2	42.5±8.2	<0.0001
BMI (kg/m^2)	25.4±3.2	37.22±6.3	<0.0001
WHR	0.87±0.07	0.94±0.07	<0.0001
WHtR	0.53±0.06	0.69±0.09	<0.0001
Systolic blood pressure (mmHg)	119.7±17.3	123.7±15.8	<0.0001
Diastolic blood pressure (mmHg)	74.3±11.1	77.8±11.0	<0.0001
Fasting glucose (mg/dL)	103.8±45.5	120.5±56.0	<0.0001
Total cholesterol (mg/dL)	178.8±37.4	182.2±34.2	0.31
Triglyceride (mg/dL)	142.8±137.7	181.3±131.2	<0.0001
LDL-c (mg/dL)	96.8±30.0	99.8±28.3	0.04
HDL-c (mg/dL)	54.6±15.5	47.9±13.0	<0.0001
hsCRP (mg/L)	4.6±9.3	8.2±8.6	<0.0001
Physical activity (steps/day)	6696±4193	5082±3490	<0.0001
Current smoking, n (%)	537 (38.9)	577 (30.9)	<0.0001
Current drinking, n (%)	855 (61.9)	1070 (57.3)	0.003
Hypertension, n (%)	323 (23.4)	695 (37.2)	<0.0001
Diabetes, n (%)	165 (11.9)	504 (27.0)	<0.0001
CVD, n (%)	39 (2.8)	75 (4.0)	0.03
